# Why data citation isn't working, and what to do about it

**DOI:** 10.1093/databa/baaa022

**Published:** 2020-05-12

**Authors:** Peter Buneman, Greig Christie, Jamie A Davies, Roza Dimitrellou, Simon D Harding, Adam J Pawson, Joanna L Sharman, Yinjun Wu

**Affiliations:** 1 School of Informatics, University of Edinburgh; 2 Information Services, University of Edinburgh; 3 Centre for Discovery Brain Sciences, University of Edinburgh; 4 Novo Nordisk Research Centre Oxford, Novo Nordisk Ltd; 5 Department of Computer and Information Science, University of Pennsylvania

## Abstract

We describe a system that automatically generates from a curated database a collection of short conventional publications—citation summaries—that describe the contents of various components of the database. The purpose of these summaries is to ensure that the contributors to the database receive appropriate credit through the currently used measures such as h-indexes. Moreover, these summaries also serve to give credit to publications and people that are cited by the database. In doing this, we need to deal with granularity—how many summaries should be generated to represent effectively the contributions to a database? We also need to deal with evolution—for how long can a given summary serve as an appropriate reference when the database is evolving? We describe a journal specifically tailored to contain these citation summaries. We also briefly discuss the limitations that the current mechanisms for recording citations place on both the process and value of data citation.

## 1 Introduction

In the general clamor to cite data, two important issues have been overlooked. First, one of the main arguments for data citation appears to be to give credit to the people responsible for creating, extracting or curating the data. But does creating and publishing data citations guarantee that these people receive credit through current bibliometric measures, such as h-indexes, impact factors, etc.? The second, and apparently completely overlooked, issue is that many databases and data sets have ‘outgoing’ citations. If an article is cited by a well-known and widely used database, should not the article and its authors also receive credit through the established bibliometric measures? Let us look at these two issues in more detail.

### 1.1 Citation of the data

There is now a large body of work on the need for data citations and how to create them. Among the reasons for citing data, giving credit is almost always prominent. For example: Data citations should facilitate givingscholarlycredit…’ (20); data citation ‘Ensures that proper credit can be given…’ (15); ‘Citing datasets …gives the researcher proper credit’ (35); ‘Data citations…provide credit for data producers’ (26). Much of the work on data citation has focused on the problem of creating something that looks like a conventional citation for data, and to this end, DataCite (14) specifies a number of fields that should be associated with a data citation. In a series of papers (6,11,12,16,37,38), it is argued that, in a general sense, some form of query is always used to extract data and that the query and the data together determine the contents of these fields. The underlying idea is that a database can be decomposed into a collection of components called views with associated citation data and that, through well-established database techniques, the citation for a query can be constructed by combining the citation structures associated with these views.

The problem is that, in order to have any effect on bibliometric measures, there has to be something like a conventional document associated with a citation, such as a paper with title, authorship, publisher, etc. If such a document is not found by ‘citation analyzers’ such as Google Scholar, which, by text mining, try to extract authorship, title, etc., a citation to the data will have no effect on bibliometric measures.

A data citation roadmap is given in (19), which describes the relationship between a citation and a landing page (the resolution of persistent identifier in the citation). The contents of that page are very close to what we propose in this paper: a method for defining, extracting, publishing and regenerating such pages. We believe, however, that such pages should be augmented with outgoing references, which we discuss next.

### 1.2 Citation by the data

Curated databases have largely replaced conventional reference works such as dictionaries, encyclopedias, almanacs, gazetteers and even textbooks. Databases have several advantages over conventional (paper) publications for the dissemination of current knowledge. Apart from ease of access and searching, they can be much larger in both size and structural complexity and they can evolve rapidly with the subject matter. The last point is especially important in molecular biology where curated databases have flourished. In its Database Issue, Nucleic Acids Research (33) lists upwards of 1000 databases. The evolution of the subject is such that few of these databases could be effectively published through conventional mechanisms. However, like conventional reference works, these databases contain abundant citations to conventional literature. Should not the authors of the cited material receive credit for being cited by a well-known database?

The need for outgoing citations is not limited to molecular biology. Even collections of digital images and numerical sensor data seldom publish ‘raw’ data; almost invariably, these data sets have been cleaned or transformed by a variety of techniques and those techniques may well warrant an outgoing reference to the relevant software or algorithms.

Again, we cannot expect citation analyzers to look into a database and extract the citations within it. If we want authors or journals to receive credit for being cited by a well-known database, there should be one or more conventional documents associated with the database, and these documents should contain a reference list for the relevant outgoing citations.

### 1.3 Outline

We claim that data citation isn't working in the sense that the creation of data citations does not per se have any effect on the current bibliometric measures. That will only happen if there are documents—rather than databases—to which the citations refer. Therefore, the problem we address in this paper is how we create a set of documents, which we call summaries, to ‘cover’ a database in that they give appropriate credit to the people who contribute to and maintain the database and that they include the appropriate citations in the database. In particular, we will be concerned with how many summaries are needed and how we deal with changes to the database. For example, do we need to create a new set of summaries for each update to the database?

In the following background section, we describe the current state of affairs with data citation in databases; we also describe a well-known curated database, the IUPHAR/BPS Guide to Pharmacology database (GtoPdb) (34) with which the authors are involved and that has been the stimulus for much work on the computational aspects of data citation. After that, we suggest some methods that might be used to generate and recommend an appropriate set of summaries to represent a database for the purposes of citation. We go into some detail on how we are generating such summaries for GtoPdb.

Although the motivation for this work was to provide bibliometric credit to the contributors to a database, the inclusion of the summaries into the ambit of on-line publishing has additional advantages of making the database and the material it cites accessible through a variety of systems. We describe an on-line journal specifically for holding these summaries. This journal could, in principle, be extended to hold a much broader class of data citations.

Finally, we describe the limitations of this approach and speculate on how one might modify or augment the structures used in bibliometrics in order to provide a more flexible approach to data citation.

**Fig. 1:**
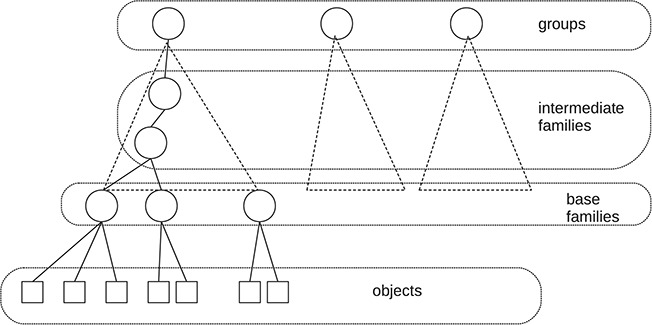
Simplified structure of the web presentation of GtoPdb.

**Fig. 2:**
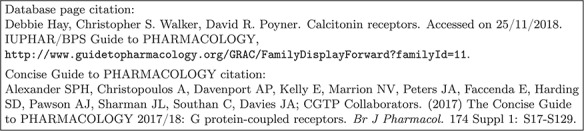
Former citation recommendations in GtoPdb.

**Fig. 3:**
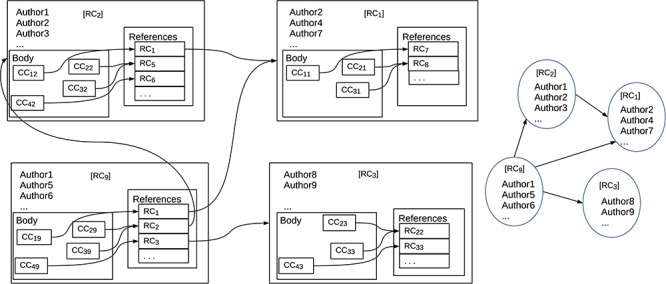
References, citations and the associated citation graph.

## 2 Background

In this discussion, we use the term database to refer to any collection of data that has some structure and that evolves over time, and we will use ‘query’ for any method of extracting data. Thus, a database may be anything from a collection of files with associated metadata to a full-blown relational database. A query may be anything from a URL or a file name to an SQL query. With this understanding, there are two notions of what data citation means. The first is a form of provenance—keeping enough information to enable one to reconstruct and verify the results of a query against the data. This may be a non-trivial task: it may require properly versioned data and careful documentation of the query and the context in which it was evaluated. See (32) for a working system that keeps complete provenance for relational database queries.

The second notion is to create something that looks like an extension of a conventional citation: a collection of attribute values such a title, authorship, date, etc. as specified in the Dublin Core (18). For example, DataCite schema (14) extends the notion of authorship and provides for optional extra fields that might be associated with geospatial data.

In (12), the computational problems of creating data citations of the second kind have been discussed. The main observations are that a citation needs to be generated for any evaluation of a query and that the citation depends both on the query and the current state of the database. It starts with the notion of a ‘view’, which is nothing more than a query or collection of queries that selects some part of the database. The technique proposed is to decompose the database into a collection of views, each with a known citation, and to compute the citation attributes for a query based on a well-known database technique of rewriting queries through views. In the simplest case, if we have a query $Q$ and a view $V$, and if we can rewrite the query through the view with a query $Q^{\prime}$, that is $Q(D) = Q^{\prime}(V(D))$ for any database $D$ conforming to some schema, then the citation associated with $V$ and the current database is an appropriate citation for the evaluation of $Q$ against the database. However, as we have already remarked, that citation will only be effective in providing credit to the authors if there is a document associated with the view.

### 2.1 Why are databases cited?

It will be helpful to speculate on why one would cite—or maybe not cite—a database that one has consulted. There are at least three distinct reasons:

- Original material. The database is the primary source for some data or analysis. One cites it because one makes use of the data in the database.
- Reference work. The database acts as the standard reference for a topic. This is where one goes for more detail. GtoPdb, for example, is often cited as the authority for the classification of compounds.
- Pass through. One uses the database, as a reference work, to find the source—perhaps some other publication—for some information. In this case, the primary citation one gives is to that publication and not to the database. Whether or not one should cite the database depends on how important or useful it was in finding the source. In this case, data citation resembles software citation. One does not normally cite software, such as a programming language compiler, that is in ubiquitous use, but one might cite a piece of software such as a sequence alignment program that helps one identify a gene.

It is clear that the first two of these reasons are perfectly good reasons for citing the database: they are no different than the reasons for citing conventional publications. In the first case, one would like to be as specific as possible: if there is a specific part of the database (a view) that is used, then the view and the people responsible for that view should be acknowledged in a citation. In the second case, one might want to cite the whole database or, again, some part of it. Thus, it can be desirable to have citations with various levels of granularity.

Let us assume that we can, as described above, associate with the database a number of views, each with prescribed authorship; that is to say that we can express, as a set of queries, parts of the database and the people who contributed to them. The obvious answer of what summaries to generate is therefore to generate a publication for each view. The problem with this solution is that there may be several hundred such views, and generating a large number of publications may not be in the best interests of the authors. In many cases, it may be possible to merge views to generate larger, more comprehensive publications with larger sets of authors, so why not continue this process and generate one publication for the entire database?

For some databases a single publication may be adequate, but in others it is not. Consider the analogous process in, say, an issue of some journal. Why not merge all the papers into a single paper and combine the authorship? This should dramatically increase the individual author’s citation counts as well as the impact factor of the journal (roughly, the number of citations divided by the number of papers in the two previous years; see (21,27).) We do not do this because it is inappropriate, if not dishonest, to give credit to someone for work they did not do. Conversely, why not subdivide individual papers into ‘minimal publishable units’, thereby increasing the number of publications of some authors? In some databases, and in GtoPdb in particular, it is possible to decompose the database with various degrees of granularity, and we need to decide which is the most appropriate. This is the main problem of concern in creating citation summaries for GtoPdb. Even from the viewpoint of outgoing citations, a single document with all outgoing references may be less appropriate than a decomposition of the database into several views. A paper that is cited several times across the database may receive more credit when the database is decomposed.

### 2.2 GtoPdb

The IUPHAR/BPS^1^ Guide to Pharmacology Database GtoPdb (34) has been a stimulus for the study of data citation in curated databases. First, it is a complex, but well-organized, relational database. The data and metadata are uniformly represented within the database. Approximately 1000 researchers from many parts of the world have contributed to the database, and it was the desire—by the curators—to give recognition to the contributors that led to some early work on data citation (11). A somewhat simplified diagram of the web presentation of GtoPdb is shown in Figure 1. As with many ontologies, it is organized into a hierarchy of families. At the top level, there is a major taxonomic division into seven groups (such as receptors, ion channels and enzymes). At the lowest level in the hierarchy, there are about 770 base, or bottom-level, families, and these families contain some 2000 ‘objects’—the drug targets described by the family hierarchy. For example, within the group ‘G protein-coupled receptors’, we find the base family ‘Calcitonin receptors’ and within that family the object ‘AMY$_1$’. The contributors associated with each object and family are stored in the database.

In addition to providing an online open-access database, the GtoPdb curators publish every 2 years a Concise Guide to Pharmacology (8). This is automatically created from the database and resembles a normal publication (a paper version is available) and is available from a well-known publisher. It consists of eight sections, one for each top-level group, and the individual sections (e.g. G protein-coupled receptors (7)) can attract 1000 citations or more in Google Scholar. While the Concise Guide itself is substantial (some 450 detailed pages), the authorship is limited to the curators, so these are the people who receive bibliometric credit. Moreover, of the 34 000 or so outgoing references in recorded the database, only $\sim $10% appear in the Concise Guide.

To reinforce the point, Figure 2 shows part of the user interface to the database before we undertook this project. Two possible citations are offered for a base family. The first is hardly satisfactory as a data citation, but even if it were, it would not be picked up by citation analyzers because there is no matching document. The second one is picked up, and attracts numerous citations, but it does not acknowledge the expertise of the three people who did the work relevant to that family and to the objects in it^2^.

### 2.3 Other databases and citation methods

We are using GtoPdb as an example, simply because it provides an ideal environment to experiment with data citation: the data and metadata are all well-represented in the database. We will look the applicability of what we develop to other databases, but we should remark that many databases already have an associated publication. In molecular biology, the database issue of Nucleic Acids Research (33) carries articles by the curators of a large number of databases. These articles typically discuss new features of the database, new content, curation techniques, etc. The authors of these papers are typically the curators or organizers of the database, and, in some cases, the only people who have worked on it so, from the standpoint of author recognition, these publications may suffice. In other cases, there are many people who have worked on the database who are not recognized in such a publication. Moreover, these publications do not contain the outgoing citations from the database, so citations by the data remain uncredited.

We should remark that most scientific publications are based on experimental data, and there are now several ‘data journals’—(1,4,31) for example—that will publish a conventional paper and also make available, in some machine-readable form, the associated experimental data, which is usually a fixed or static data set. In these journals, a citation to the paper will usually serve as a satisfactory citation to the data. Our focus is on the very large number of evolving databases with some degree of curation, which may have aggregated the data from individual publications or may contain data that were not collected for a specific experiment, and for which there is currently no effective system for citation of parts of—or views of—the data. Our goal is to generate the appropriate publications from the data.

### 2.4 Citations, references and reference annotation

Before going further, and in order to understand data citation, we need to look at the structure of a standard citation. Consider what one might put in the text of a paper, something like Emily Brontë, Wuthering heights, Ignatius Press, 2008. pp 21–25. There are two parts to this. First, Emily Brontë, Wuthering heights, Ignatius Press, 2008, is determined entirely by the document being cited. We shall call this a ‘reference’. The second, pp. 21–25 is some additional information that may help the reader find the relevant information. We shall call this ‘reference annotation’. We shall call the combination of these a ‘citation’. Note that a reference is determined when the referenced document is published, but the reference annotation is determined when the citing document document is ^3^.

Figure 3 shows the distinction. The diagram on the left shows the internal structures of a set of documents: how references are associated with documents and how documents are associated with each other. The nodes represent publications and the arrows represent references from one publication to another. Inside the nodes, there are boxes labeled ‘CC...’ that are citations in context, i.e. they contain both reference and reference annotation; the boxes labeled ‘RC...’ contain just references. We stress that this is a ‘logical’ view of the structure of a document; how the nodes are textually represented depends on the document style. The nodes also contain information such as title, authorship, publisher, etc. The arrows show how references link publications. While the arrows normally point backwards in time, cycles are not excluded. The diagram on the right shows a simpler graph in which reference annotation is missing from the nodes, though other information such as title and authorship is maintained. This graph is what is normally called a ‘citation graph’. All well-known bibliometric measures such as h-indexes and impact factors are based on a citation graph. Thus, these measures cannot take account of reference annotation.

The distinction between reference and reference annotation may seem somewhat pedantic, especially when many papers, such as this, have little or no reference annotation. However, when it comes to data citation, the distinction appears to be important. The DataCite schema (14) has optional geolocation data (e.g. a bounding box for geospatial data) that, like page numbers, serve to locate the data of interest and should be treated as reference annotation. Another important use of reference annotation arises when what is being cited changes over time. In this case, it is common practice to provide, as part of reference annotation, the time at which a database is cited or the version being cited. In the case of a web page it is customary to give a ‘retrieved on’ date.

When it comes to authorship, the normal assumption is that authorship is part of the reference and not reference annotation. However, the DataCite schema also has provision for two kinds of author: creators, ‘the main researchers involved in producing the data...’, and contributors, ‘the institution or person responsible for collecting, managing, distributing...’ the resource. The terminology does not align well with the terminology we are using here, but the observation is that if, as in GtoPdb, the creators vary with the part of the database being cited, there are two possibilities. The first is to treat creators as reference data, which means that we need to create a summary for each part (view) of the database being cited. This is what we do in this paper. However, another possibility is that we treat the creators like page numbers and bounding boxes as reference annotation. If we do this, then they do not appear in the citation graph and will not be found by citation analyzers. This is a problem with the approaches to data citation in (16,37) and possibly (32), though the prime purpose of (32) is to provide accurate provenance rather than ascribe credit. We discuss this further in the conclusions.

## 3 Creating database summaries

We now turn to the main challenge addressed by this paper: to create a set of skeletal publications which, for the purposes of bibliometric measures, properly represents the database. That is we want to create a set of summaries that will be recognized by citation analyzers and will be represented as additional nodes in the appropriate citation graph. We will call these ‘database summaries’. We will be concerned with three issues: what summaries to generate, the contents of these summaries and how to cope with change in the database. It must be remembered that the prime function of these summaries is to be ‘read’ by citation analyzers and not by people. Nevertheless, people may well land on these summaries if they are following citation links, so we need to be sure that it is clear why they are there and how to get to the relevant parts of the database that they represent.

There will be no general method of finding which summaries to generate automatically. At the very least, we are going to require the authors’ permissions to create summaries that they ‘authored’. More importantly, database curators will usually have some idea of who was responsible for what part of the database. So at best, we can expect to come up with some recommendations of what these summaries are and, in the likely event that there are several possibilities for generating sets of summaries, to provide measures that can be used to compare these possibilities.

### 3.1 Finding the views

Let us assume that we have no guidance on what these summaries will be, and let us start by trying to create these based on authorship. If the authors/contributors are listed in the database, there will almost certainly be one or more tables with a scheme $C(A,T)$ that express the fact that author in $A$ has contributed to ‘thing’ in $T$ in the database. In the case of GtoPdb, a ‘thing’ could be a family or object. One possibility is to associate a view, and a summary for that view, with each element of $T$. However, as pointed out in the section on Why Databases are Cited, this may generate far too many summaries, and we may want to find coarser clusters of authors that represent the collaboration of authors and that may produce a better basis for generating the summaries. A possible way to generate the summaries is to cluster the things by their similarities of authorship. For this, we need a clustering method that does not require *a priori* knowledge of the number of clusters to be generated. We decided to experiment with a clustering algorithm that produces a partition of the set of things and does not require the size of that partition as input.

### 3.2 Correlation clustering

**Fig. 4:**
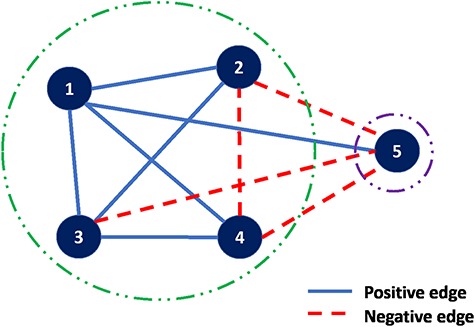
Correlation clustering example.

The details of ‘correlation clustering’ are described in (10). The input is an undirected graph in which the edges are positive or negative. When sets are associated with the nodes, the sign of an edge can be determined by any well-defined similarity measure such as Jaccard similarity. The goal is to partition the input graph into multiple clusterings of the nodes such that in each cluster, as far as possible, any positive edge is in one cluster and any negative edge connects two distinct clusters. An example of such graph is shown in Figure 4, a complete graph on five vertices and edges labeled positive or negative. A good clustering is $\{\{1,2,3,4\},\{5\}\}$ with just one misplaced negative edge, $(2,4),$ and one misplaced positive edge, $(1,5)$.

In order to extract a positive/negative edge-labeled graph from we compute for each pair $(t_1, t_2)$ of things, the Jaccard distance between the author sets of $t_1$ and $t_2$; we then used a threshold and labeled any pair whose distance was less than the threshold as positive; otherwise it was labeled negative.

Using a threshold of 0.5, we applied correlation clustering to a version of the database with a total of 3820 objects and families to obtain 244 clusters. This reduction is somewhat misleading because only 640 of these families and objects have contributors. Since (see Section on Application to GtoPdb) the curators decided that they wanted only to generate summaries for families, the interesting questions were how many clusters would contain more than one family and would these clusters agree with the hierarchy, suggesting summaries for higher level families? After a certain amount of cleaning prompted partly by initial runs of the algorithm, 14 such clusters were produced of which 6 correctly identified superfamilies (a family and all its subfamilies) in the hierarchy. Moreover, all of these were subsequently assigned summaries by the curators. The other 8 were groups of families that were close in the hierarchy as follows. Further details are given in Appendix A.

(i) Five were sets of sibling families but not complete sets of siblings in the existing hierarchy.
(ii) One was a cluster that contains a parent family and a child family in the hierarchy but not all the children of the parent.
(iii) One was a complete set of siblings, not the parent, but two ‘aunts’.
(iv) There are a few cases in which an object has more than one parent. This can cause an anomalous cluster.

**Fig. 5:**
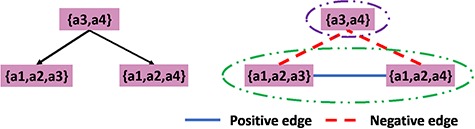
Examples of the use of correlation clustering algorithm in the hierarchical database; the left-hand side represent the hierarchical structure of the example database while the right-hand side shows the clustering results.

The left part of Figure 5 shows why correlation clustering may not find all the superfamilies. Suppose we have a parent node with author set $\{a3,a4\}$ and two child nodes with author sets $\{a1,a2,a3\}$ and $\{a1,a2,a3\}$ and $\{a1,a2,a4\}$, respectively. Using a threshold of 0.5 on the Jaccard distance, we get the result shown in Figure 5. Since there are more negative edges than positive edges, we cannot merge them as a cluster. However, the intuition is that it is reasonable to cluster all three nodes. This disagreement is because the clustering algorithm is based on the difference between each pair of the nodes in the graph rather than on the combined authorships of a set of nodes that potentially constitute a cluster. This motivates us to propose a measure of how disparate the combined authorship will be. Also with such a measure, we can annotate each node in the hierarchy with a numerical value to indicate to the curators whether it is appropriate to have an associated summary.

### 3.3 Stress measures

If the authorship associated with a family is identical with the authorship of each of the objects in that family, presumably the authors should be happy for a single summary to be generated for the family and all of its objects. We need to measure the degree of unhappiness or ‘stress’ in a proposed merger of authorships. Clustering based on Jaccard distance, as we have seen, does not directly provide such a measure.

We shall deal with sets of authors rather than lists. (As far as we are aware, bibliometric measures do not take account of author order.) If we have just two sets $X$ and $Y$ of authors, the Jaccard distance is defined to be $1 - \frac{\vert X\cap Y \vert }{\vert X\cup Y \vert }$. We can easily extend this measure to a set—or multiset—$T$ of sets of authors by defining $\textsf{stress}(T) = 1 -\frac{\vert \bigcap T \vert }{\vert \bigcup T \vert }$. This measure, like the Jaccard distance, varies between 0 and 1. It also has the property of being ‘monotonic’. That is, if $S \subseteq T$ then $\textsf{stress}(S)\leq \textsf{stress}(T)$.

However, this measure may be seen as too Draconian: $\frac{\vert \bigcap T \vert }{\vert \bigcup T \vert }$ is the number of authors who contributed to all members of $T$ divided by the number of authors who contributed to some member of $T$. If all of several authors contributed to all but one of the members of T, this does not reduce the stress. A somewhat less strict measure can be obtained as follows. We define $F(T,i)$ to be the set of authors who have contributed to exactly $i$ of the members of $T$, that is $F(T,i) = \{a\in \bigcup T\ \mid \ \vert \{X\in T \mid a\in X\} \vert = i\}$. Note that $F(T,1), F(T,2), \ldots , F(T, |T|)$ is a partition of $\bigcup T$, that $\vert \bigcap T \vert = \vert F(T,\vert T \vert ) \vert $ and that $\vert \bigcup T \vert = \Sigma _{i=1}^{\vert T \vert }\vert F(T, i) \vert $

Now, let $\alpha (n,i)$ be a real-valued function defined whenever $1\leq i\leq n$ and consider the stress function

$$\begin{equation*}\textsf{stress}_{\alpha}(T) = 1 - \frac{\sum\limits_{i=1}^{\vert T \vert}\alpha(\vert T \vert,i)\vert F(T,i) \vert}{\vert \bigcup{T} \vert}\end{equation*}$$

If we define $\alpha (n,i) = 1$ if $i=n$ and $0$; otherwise, $\textsf{stress}_{\alpha }(T) = \textsf{stress}(T)$, the generalization of Jaccard distance. For general $\alpha $, $\textsf{stress}_{\alpha }$ is not monotonic. However, we have the following:

**Prop**. If $\alpha (n,i) \leq \alpha (n+1,i+1)$ and $\alpha (n,i) \leq \alpha (n+1,i)$ whenever $1\leq i \leq n$, $\textsf{stress}_{\alpha }$ is monotonic.

Examples of such an $\alpha (n,i)$ are $\frac{1}{n+1-i}$ and $2^{(i-n)}$. With the second function and $T=\{ \{b,c,d\}, \{a,c,d\}, \{a,b,d\}, \{a,b,c\}\}$, $\textsf{stress}_{\alpha }(T) = 0.5$.

### 3.4 Application to GtoPdb

Each node (family or object) in the GtoPdb hierarchy (Figure 1) could, in principle, have an associated citation summary. However, the curators felt that generating over a thousand summaries was inappropriate and probably hard to maintain. Some kind of aggregation is appropriate, but how should this be done? To start with, the relationship between the hierarchy and the desired summaries is not straightforward. One might assume that each summary will serve as a citation for a node and all of its descendants, but it is perfectly possible to have a summary associated with a family and another summary associated with one or more of its subfamilies. In fact, as seen in Figure 2, the reader is offered two possible citations: one for the family or object described by the web page and one for the top-level group for that family.

**Fig. 6:**
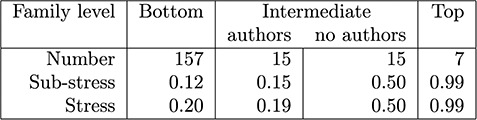
Stress statistics for GtoPdb families

The stress measurements for families grouped by levels (Figure 1) are shown in Figure 6. Not all families or objects have listed authors, and the number is quite variable (maximum of 33). For each family we compute its sub-stress – the stress of the authorships of all the descendant families; we also compute its stress – the stress obtained by adding in the authorship of the family itself. The means of these figures are shown in the table. 

Early on in the discussions of how to generate summaries, the curators decided to avoid, if possible, generating citation summaries for individual objects and to start with the bottom-level families. After a certain amount of data cleaning, about two-third of these families had a sub-stress of 0 and the figures in this column are skewed by a few outliers that required some resolution. At the top level groupings, authorship is not recorded (credit is given to the curators), but the stress indicates that recording authorship at this level would be a bad idea in any case.

The intermediate values are the most interesting. When an intermediate level family has an author set, the stress is relatively low, indicating that it may be appropriate to merge the citation summaries upwards; however, those that have no authors require individual attention. For example, authorship could be taken as the union of the authorship of subfamilies, or it could be given to the curators.

Ultimately, it is up to the curators and authors to decide on which citation summaries to generate. A procedure that was effective in helping the curators decide on how to group families and objects for the purpose of publishing summaries was based on this analysis. We compute stress bottom-up on the hierarchy and annotate it as follows: suppose $N$ is a node and $N_1,\ldots , N_k$ are its children, and use $N$ ambiguously to refer to the node and to its set of authors, and we have already computed $\textsf{stress}(N_i)$ for $1\leq i\leq k$.

(i) if *N* has no authors: (not all nodes have authors)
  (a) if all the stresses $\textsf{stress}(N_i)$ for $1\leq i\leq k$ are low and also $\textsf{stress}(\bigcup _{1\leq i\leq k}N_i)$ is also low, consider generating a summary for $N$ and its descendants;
  (b) otherwise, generate summaries for $N_1,\ldots , N_k$ if they are not already generated
(ii) else if $N$ has authors
  (a) if $\textsf{stress}(N\cup \{N_i \mid 1\leq i\leq k\})$ is low, generate one summary for $N$ and its descendants,
  (b) else generate a summary for each of $N$ and $N_1,\ldots , N_k$.

Although this is somewhat underspecified, the interesting cases are 1a and 2b. We can flag these cases as deserving some review by the curators; the others are obvious. Doing this substantially reduced the number of problem cases to about 30. These were nearly all outliers in the bottom-level families and in the intermediate families with authors. Ultimately, the set of families for summaries was reduced to 138 with 15 being higher-level.

To return to correlation clustering, the use of correlation clustering algorithm over GtoPdb produces clusters in which $\sim $77% of the clusters have 0 stress while $\sim $90% have very low stress (¡0.3). So roughly speaking, correlation clustering agreed with the curators’ decisions in ¿90% of the clusters. The problem is that correlation clustering did not directly catch the 10% problem cases. Because we have an existing hierarchy, the stress measure was somewhat more informative.

### 3.5 The contents of a citation summary

First and foremost, the structure of the summaries should be such that they are recognized by citation analyzers. Google Scholar (3) makes it clear what is needed for a document to be recognized: a PDF file in a website that resembles a conventional paper, with a title, list of authors, contents and references.

In addition, we want to make it clear that the summary is a proxy for some view of a database. Thus, an adequate list contains

(i) a title, which contains the version of the database (see below);
(ii) an author list with affiliations;
(iii) a short descriptive text. If one can be extracted from the database, this will serve as an abstract;
(iv) links (URLs) to the database itself—reader landing on this summary should be steered immediately to the database;
(v) the ‘publisher’— this may be the organization that curates the database, but it could also be that of the organization that publishes the citation summaries.
(vi) a statement of the ‘rationale’ for the summary: to create a node in citation graphs and register the database in bibliometric measures;
(vii) a reference list (the outgoing citations); and
(viii) a digital object identifier. This is required by Online Journal Systems (see below).

**Fig. 7:**
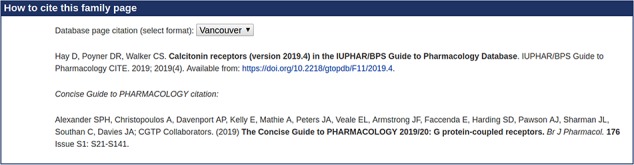
A screenshot of the citations on a GtoPdb web page.

An example summary is shown in Appendix B, for which the citation (24) is shown in Figure 7, a screenshot of the relevant web page of the GtoPdb user interface. This should be compared with Figure 2. We have provided a standard drop-down menu that provides the citation in various bibliographic formats, making it easy for authors to copy a citation into a paper. It should be re-emphasized that the primary function of these summaries is to be read by citation analyzers and not by people. Nevertheless, people following conventional citations will inevitably ‘land’ on one of these documents. It is important that the reader is redirected to the relevant web pages of the database; so all relevant links back to the database are provided in the summary (point 4 above.) Moreover, the web page to which the DOI resolves contains, most prominently, a link back to the database.

In our analysis of GtoPdb, we created 138 summaries with an average of 7.5 authors per summary. The reference list in each summary contains references for the relevant family and all its descendants. On average, there are 166 outgoing references per summary (see Appendix A).

Such a summary will provide conventional reference for the database view and will serve the purposes of bibliometric credit. But should a citation to the database also contain some additional reference annotation (see section on Citations, References and Reference Annotation)? We strongly believe that some form of provenance is appropriate. In the case of a GtoPdb web page, it should be the URL of that page together with a version number; in other cases, it could be a pointer to a record of whatever process was used to extract the data (32). It could also contain some of the fields in the DataCite schema that are appropriate for reference (as opposed to reference annotation). However, this should all be done in such a way as not to confuse citation analyzers.

### 3.6 Publishing the summaries

Although Google Scholar (3) indicates that it is enough to place the summaries in an accessible website, it also recommends the use of institutional repositories or established journal hosting services, as these sites export data in a form that Google Scholar and other bibliometric software can read.

For some time, the University of Edinburgh Information Services, which manages the library and university-wide computing infrastructure, has provided a stable host for web presentation of GtoPdb, and it was natural to ask if they would also agree to provide web space for the summaries. Their suggestion was to use the journal hosting service that the library provides. The services use the Open Journal Systems (OJS) (36) software, which is also recommended by Google Scholar.

We mentioned in the Introduction that a curated database resembles closely an ongoing reference work more than a journal. It is nevertheless reasonable to have a journal dedicated to new developments in a data collection (much as the database issue of NAR is dedicated on a wider scale to developments in molecular biology databases). Thus, a possible title for the journal could be ‘What’s new in GtoPdb?’ where one would place articles describing modifications and new materials in the database, but this might be interpreted as a rather coarse-grained description of the evolution of the database. Instead, we adopted the more direct title of ‘IUPHAR/BPS Guide to Pharmacology CITE’^4^ .

Once created, the summaries can be uploaded manually or automatically, provided one provides the appropriate XML metadata. Since we are in any case generating both PDF and HTML versions of the summaries, providing the additional XML metadata for automatic upload is a relatively straightforward task.

In addition to providing a stable repository for citation summaries, and readable metadata, there are some other advantages to using OJS. As a publishing platform with built-in interoperability features, OJS makes articles discoverable and accessible across a range of systems and services. It is optimized for Google Scholar indexing, it supports direct metadata deposits to PubMed, the Directory of Open Access Journals (17) and institutional repositories, and it works well with commercial abstracting and indexing services like Scopus and Web of Science. OJS also has support for richer metadata, including Open Research and Contributor IDs, Funder Registry IDs and machine-readable licensing information (5), and it can be used to assign digital object identifiers (DOIs) to articles and to deposit article metadata and associated references directly to DOI registration agencies, in our case Crossref (13). Being able to use DOIs in turn gives us access to a range of DOI-based tools and services and makes article metadata more widely available through Crossref.

OJS also has its own REST API for access to search and filter article metadata, as well as an OAI-PMH endpoint, through which article metadata can be extracted in a variety of formats, including MARC21, RFC1807, JATS, MARC and Dublin Core (9,18,28,30). In this way, we can recall the metadata we have provided. This is important because it reduces the amount of additional information we have to store in GtoPdb itself. For example, additional tables would be necessary in GtoPdb to record the history of publication of summaries and the change of authorship. This is not needed if we can retrieve it from OJS metadata (see below).

### 3.7 Dealing with change

Having generated a set of summaries, a critical question is how often do we need to change them? Curated databases vary in how often they are updated and how often new versions are released. In GtoPdb changes are made daily, but a public version is released a few times a year. How often do we need to update these summaries? Apart from other considerations, to generate new summaries on each update to the database, or even on each public release of the database, could create a large, possibly unmanageable, number of summaries. There are two observations that simplify the situation. First, the summaries are generated from views of the data, and an update to the database may not change a specific view; in other words, views change more slowly than the whole database. Second, the purpose of these summaries is to represent authorship and outgoing citations in a citation graph. If these do not change and other changes in the view are deemed insignificant then there is no need to change the summary.

In what we have developed for GtoPdb, each summary is given a version number associated with the view. Moreover, in order for citation analyzers to distinguish successive summaries, the version number is included in the title (the summary is also assigned a new DOI). In order to decide when to generate a new summary, we need to keep a record four things: (i) the version number of the database that was used to generate the existing (most recent) summary, (ii) the authorship for that summary, (iii) the outgoing references for that summary and (iv) an encoding of the view of the database covered by the citation. We could modify the database to keep this information, but one of the advantages of having the summaries managed by OJS is that the metadata is accessible, and all of these can easily be accommodated in the metadata, for (ii), (iii) and (iv) are nothing more than lists—or nested lists—of internal identifiers in the GtoPdb.

What this means is that publishing these summaries requires almost no change to the database itself. Whether to trigger the publication of a new summary can be determined from the contents of the database and the OJS metadata. Of course, the curators have option of generating a new summary for other reasons such as a substantial change in the contents of the relevant view.

In general, the treatment of time in data citation poses a number of challenges. In conventional citations, the time of publication is generally regarded as part of the citation. This is reasonable because we regard summaries as fixed once they have been published. However, databases are labile and do not have a fixed time of publication, so the time at which we query the data is an essential part of the citation. This view leads, for example, to citations of the form J.Doe. How to cite data. http://citingstuff.org/datacitation. Retrieved July 2018. Is Retrieved July 2018 part of the reference or is it reference annotation? If the contents of the link are subject to substantial change, then we should regard each version as an independent publication and the retrieval time is part of the reference. In this case, it would be better if the publishers were to provide a version number^5^ so that the time of retrieval is unnecessary.

On the other hand, we could regard the database (in the case of a web page the sequence of all versions) as a single evolving object, in which case the retrieval time or version number is part of the reference annotation. In this context, it is worth remarking that many data sets have a temporal as well as a spatial dimensions. MODIS (25) is an example: it contains repeated scans of the earth’s surface, and a temporal interval would be as important as, say, a bounding box in any reference annotation. While geo-location, such as a latitude/longitude bounding box, is a recommended property of a data citation in (14), there is no mention of any temporal information that might be appropriate to a longitudinal (sic) data analysis^6^ .

## 4 Conclusions: can data citation give credit?

We have shown how to generate a set of documents that act as proxies for a database and can be absorbed into current citation graphs by the various systems that perform bibliometric analyses. GtoPdb presents an ideal example of a database in which it is appropriate to generate several documents to represent the database in order to make sure that both authorship and outgoing citations are recognized by these systems. We found that, unsurprisingly, clustering the database by authorship produced clusters that agreed well with the family groupings that are already present in the database, and that this provides the basis for a representative set of documents.

### 4.1 Other databases

We have attempted to deal with two issues in this paper: first, we have shown how to create a system for publishing and maintaining citation summaries so that authorship—both of the contributors to the database and of those publications upon which the database is built—is recognized by citation analyzers. The second was how to achieve a satisfactory set of publications that ‘cover’ the database and assign authorship to the relevant parts (views) of the database.

It is, of course, for the curators of other databases to decide how credit should be assigned to those who have contributed to the content or maintenance of the database, but there is little doubt that if we are to make databases first-class publications, then citations from the database should be recognized. Some set of citation summaries is therefore appropriate for any such database, and we have shown how it is relatively straightforward to publish these in an appropriate journal. Much of the code we developed for this is specific to GtoPdb, but the authors are happy to share any of this, in particular the code that generates bulk uploads for OJS. Once this is done, we believe that creating new summaries as described in the section on Dealing with Change is straightforward.

The second question is whether the techniques we used for finding a satisfactory set of views for the purpose of creating citation summaries is more widely applicable. We do not have an answer to this. Curated databases typically keep an internal record of who has contributed to what part of the database, and, in some cases, this is available in the published data or metadata. We looked at two examples, Online Mendelian Inheritance in Man (23) and Experimental Factor Ontology (EFO) (29) are two such examples. In the case of EFO, we tried correlation clustering, and while we obtained a satisfactory clustering, the ‘authorship’ for the clusters was typically small—one to three people—and, unlike GtoPdb, there was no obvious association of expertise with components of the database. We also tried conventional hierarchical clustering using stress measure to compute merges of clusters. This did not yield an interesting hierarchy, again confirming that, if there is a hierarchical structure in EFO, it is not well correlated with contributor expertise. Given that the number of people is an order of magnitude less than that associated with GtoPdb, then an appropriate strategy might be to create a citation summary for each release of the database. We should emphasize that this is a recommendation based on little understanding or the internal structure of EFO and no knowledge of their operating procedures. However, it would be straightforward for this or any similar organization to publish citation summaries for the whole database; the main problem to be addressed would be the frequency of publication.

More generally, the authors would be interested to know of other databases in which the expertise of the contributors is varied and associated with specific views of the database. In such cases, an analysis using clustering and stress measures is likely to be appropriate.

One of the unforeseen benefits of creating summaries was to bring GtoPdb into the scope of online publishing so that both the database and the material cited by the database is recognized by the numerous systems (section on Publishing the Summaries) that have been developed for conventional publications. One obvious and useful thing we could do is to change the name of the ‘IUPHAR/BPS Guide to Pharmacology CITE’ journal to the ‘Journal of Database Citations’ and allow summaries, which follow the the general guidelines we have described in this paper, to be submitted by the curators of other databases. However, we felt it better to walk before we run and to test the current system for a period before expanding its scope.

### 4.2 Initial experience

Since the submission of the first draft of this paper ($\sim $10 weeks), we have been able to collect a small amount of preliminary data.

*Contributor benefit*. The contributors were canvassed before we published the first abstracts. There were no objections to the details (e.g. to re-ordering and aggregating authorship) and there was general approval for what we are doing. Some of the summaries have already been lodged in ResearchGate (2) and in university repositories—presumably by academics who want conventional evidence of their research outputs. Also, contributors to the database often cite their own papers, so these citations are now registering in bibliometric measures.

*Google Scholar*. In this limited period, we have so far had five incoming citations to the summaries. We cannot expect many more because these citations will only come from papers that have been ‘turned around’ in a few weeks. To be realistic, we need at least a year to see how well the summaries work. The outgoing citations are mostly recorded. However, Google Scholar is, annoyingly, conflating some of the summaries and forgetting others. It would appear that the summaries are regarded as ‘versions’ of the same paper if their titles are roughly similar by some application of machine learning. It is not clear whether the process takes into account authorship, content or the DOI.

*Semantic Scholar*. Although slower on the uptake, this appears to have recorded accurately all of the summaries and has registered seven incoming citations (only a partial overlap with those recorded by Google Scholar). There is no conflation of papers. All the outgoing citations appear to have been recorded.

New version. Also in this period, a new version of the database was released. This resulted in six new summaries and a new issue of the journal. See Appendix A. We hope to be able to provide some general code that will interact with the OJS API that will both recommend which new summaries to generate and submit the chosen ones in the appropriate format.

### 4.3 Limitations of the approach

While generating citation summaries has the desired effect of representing the database within current citation graphs, we do not regard it as an ideal solution. First, looking at the properties proposed in the DataCite schema, even in the ‘mandatory’ category, there is a property, Resource Type, that does not align with the fields in conventional citations. The schema also proposes Creator (mandatory) and Contributor (recommended) as classes of people with responsibility for the data. Whether they should be combined and treated as conventional authors in a conventional citation is not clear. Then, as we observed in the section on Citations, References and Reference Annotation, there are properties such as geo-location that may be of help—like page numbers—in finding the relevant data, or properties like format that may be of help in interpreting it. These fall into the category of reference annotation and will definitely not be represented in the citation graph.

In (16,37), techniques have been described and implemented for creating data citations from queries, in particular, the query can determine the set of authors. The question this raises is whether to treat the authorship as part of the reference or as part of the reference annotation. If the authorship is to be captured by current bibliometric measures, then it has to appear in the citation graph and be part of the reference. This would require the construction of a document for any new set of authors produced by a query. That is, we would need to create new documents, as they are needed, from queries, and the number of such documents could be exponentially large in the number of possible authors. It is unlikely that this form of *post hoc* document generation would be manageable.

A related issue concerns ‘nanopublications’ (22). In which individual facts are turned into publications. An RDF version of some of the basic assertions in GtoPdb already exists, and it would be a trivial matter to generate some 350 000 nanopublications from one version of the database. But would having a huge number of publications each, probably, with very few or zero citations benefit the authors (see the discussion of minimum publishable units in the section on Why are databases cited?) on minimal publishable units)? Moreover, where would we place outgoing references? An alternative would be to make the nanopublication part of reference annotation to citable units constructed, perhaps, as we have decribed in this paper. The problem is that this reference annotation is not normally picked up by current citation analyzers.

We are left with the conclusion that, while bibliometric measures are taken from citation graphs in their current form, it is going to be difficult or impossible to make full use of data citation in the assignment of credit. The best we can do is to provide some approximate representation of databases in the citation graph, and this is what we have attempted to do in this paper. To use the full potential of data citation in citation analysis, a new form of citation graph is needed that is tailored to the complex and dynamic use of databases. For this, further research is needed.

## Figures and Tables

**Fig. 8:**
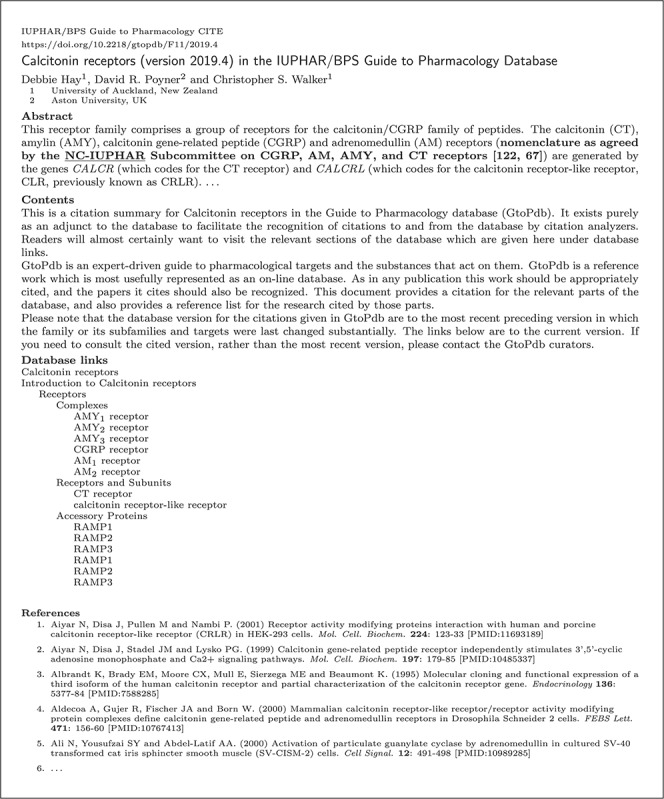
Figure 8 Shows a heavily redacted (for reasons of space) summary that has been generated from GtoPdb. This is a paper (24) in the OJS Journal GtoPdb CITE. Electronic versions of this summary should provide working hypertext links to appropriate sections of the database and cited material.

## Footnotes

1 International Union of Basic and Clinical Pharmacology/British Pharmacology Society

2 The Concise Guide does acknowledge ‘CGTP collaborators’, but provides only a partial list, and these do not appear to be picked up as authors by citation analyzers.

3 It may be that a better terminology is needed as the standard distinction between a citation and a reference appears to be that that citations go in the body of a paper while references go at the end in a bibliography, or possibly in footnotes. (See, for example, McAdoo, T. References versus citations, http://blog.apastyle.org/apastyle/2017/09/References-versus-citations retrieved December 2018; and Eaton, S.E. What’s the difference between a citation and a reference? https://drsaraheaton.wordpress.com/2013/10/18/whats-the-difference-between-a-citation-and-a-reference/ retrieved December 2018.)

4
http://journals.ed.ac.uk/gtopdb-cite

5 As is done, for example in WWW consortium publishing.

6 An oversight that appears to have been corrected in the latest version of the DataCite recommendations.

## A.

### A **Appendix 1** Further details

The home page of the GtoPdb is found at https://www.guidetopharmacology.org/. This, of course, provides the latest state of the database and not the version from which the statistics and other details are recorded in this paper. Note that on many pages the contributors for that page are recorded. They should be compared with the authors listed in the citation. Also, perhaps because there are no contributors to a web page, there is no citation or summary for some pages. Instead, a URL of the page itself is provided.

The OJS journal is found at http://journals.ed.ac.uk/gtopdb-cite/. In our first round of publishing, we generated 138 summaries, and these are recorded in the first issue (2019.4) of the journal. There are some structures (e.g. ligands) for which summaries might be appropriate but require further organization to produce them. Any family was considered for inclusion in a summary if either it had contributors recorded in the database or it had a descendant object or family with recorded contributors. The published summaries in the first issue cover a total of 1417 objects and families in the database.

Following the next release (2019.5) of the database, there is a new version of the OJS journal, which contains six new summaries. Of these, four were new entries and two were delayed from the first issue while database issues involving the contributorship were straightened out.

Outgoing references. Because our main concern is for credit to be given to the contributors, we have largely ignored outgoing references in our grouping of the objects and families into summaries. However, the summaries contain all the outgoing references for their component objects and families. The summaries have a total of 22 900 outgoing references, of which 22 174 are distinct. Thus, the majority of these references are cited by just one of the summaries. Only eight references are cited by more than four summaries. Whether clustering by outgoing references would produce similar aggregations into summaries requires further research; however, we can say that most of the active references in the database are now being recorded by citation analyzers.

Clustering anomalies. In Section 3.1, we gave four examples of how clusters that were either inconsistent with the existing hierarchy or suggested superfamiles that did not accord with the curators’ opinions. The following show some more details of these anomalies, which have persisted into the current database. To see these, use https://www.guidetopharmacology.org/GRAC/FamilyDisplayForward?familyId=〈number〉 or https://www.guidetopharmacology.org/GRAC/ObjectDisplayForward?objectId=〈number〉 where <number> is the number of a family or object as appropriate.

$\bullet $ Sets of sibling families but not complete sets of siblings in the existing hierarchy. See families 446 and 447. The contributor sets are $\{A,B,C\}$ and $\{B,C\}$, respectively. There is no superfamily to include these alone, nor do the curators think there should be one.
$\bullet $ Clusters that contain a parent family and a child family in the hierarchy but not all the children of the parent. For example, {787, 777 776}. The parent, 776 is missing its child 778.
$\bullet $ A complete set of siblings, not the parent, but two ‘aunts’. {279, 253, 278}. The parent of 253 and 239 has no contributors.
$\bullet $ An object has more than one parent. See object 1399.

When an object is on more than one path, there is a ‘main’ path in the hierarchy to that object. The citation for that object is the summary that appears on that path. However, the object is listed in the summary of any family for which it is a descendant.
